# The Self-Assembly of a Cyclic Lipopeptides Mixture Secreted by a *B. megaterium* Strain and Its Implications on Activity against a Sensitive *Bacillus* Species

**DOI:** 10.1371/journal.pone.0097261

**Published:** 2014-05-09

**Authors:** Manuel T. Pueyo, Bruna A. Mutafci, Marco A. Soto-Arriaza, Paolo Di Mascio, Ana M. Carmona-Ribeiro

**Affiliations:** 1 Departamento de Bioquímica, Instituto de Química, Universidade de São Paulo, São Paulo SP, Brazil; 2 Departamento de Química-Física, Facultad de Química, Pontificia Universidad Católica de Chile, Macul, Santiago, Chile; Cornell University, United States of America

## Abstract

Cyclic lipopeptides are produced by a soil *Bacillus megaterium* strain and several other *Bacillus* species. In this work, they are detected both in the *Bacillus* intact cells and the cells culture medium by MALDI-TOF mass spectrometry. The cyclic lipopeptides self-assemble in water media producing negatively charged and large aggregates (300–800 nm of mean hydrodynamic radius) as evaluated by dynamic light scattering and zeta-potential analysis. The aggregate size depends on pH and ionic strength. However, it is not affected by changes in the osmolarity of the outer medium suggesting the absence of an internal aqueous compartment despite the occurrence of low molecular weight phospholipids in their composition as determined from inorganic phosphorus analysis. The activity against a sensitive *Bacillus cereus* strain was evaluated from inhibition halos and *B. cereus* lysis. Essential features determining the antibiotic activity on susceptible *Bacillus cereus* cells are the preserved cyclic moiety conferring cyclic lipopeptides resistance to proteases and the medium pH. The aggregates are inactive per se at the pH of the culture medium which is around 6 or below. The knock out of the sensitive cells only takes place when the aggregates are disassembled due to a high negative charge at pH above 6.

## Introduction

The genus *Bacillus* includes Gram positive, phylum Firmicutes, class *Bacilli*, low G+C species capable of colonizing a broad range of environments. Although this may be a consequence of its remarkable genomic and metabolic diversity, human organisms seem not to be suited to host these bacteria. Description of spore-forming *Bacillus subtilis* strains as human gut commensals, do not change noticeably this picture [1]. Yet, a few *Bacillus* species are known as human pathogens. Among these are *Bacillus anthracis* and *Bacillus cereus*, species that are closely related [2]. Some evidences suggest that *B. antracis* was originally a soil microorganism [3], [4], which probably evolved from a *B. cereus* ancestor through horizontal transfer of plasmid-encoded virulence factors [5]. *Bacillus thuringiensis*, a species pathogenic to insects, is also related to *B. anthracis* and to *B. cereus* [6]. Entry into the digestive system is a fundamental event in animal and insect pathogenesis brought by *Bacillus*. To accomplish this, the spore forming capacity seems to be of crucial importance. Broadly speaking, the genus *Bacillus* is not pathogenic to humans. This is in sharp contrast with other Gram (+), Firmicutes, low G+C bacteria belonging to the genus *Staphylococcus*. They are harmful to the humans in its vast majority. *Bacillus subtilis* and *Staphylococcus aureus*, largely different in morphology, are highly similar concerning gene regulation and protein sequences [7] although the repertoire of substances secreted by these microorganisms may be considerably divergent. The nature of these substances and the properties of the outer cellular surface are in a straightforward relationship with the survival in an environment which can be, among others, living organisms such as plants, insects and animals, including humans. In this regard, *Pseudomonas*, a gammaproteobacteria genus encompassing almost 200 Gram negative species, also displays a remarkable plasticity in terms of niche occupation. For example, *Pseudomonas aeruginosa* is an opportunistic soil bacterium possessing a high degree of human pathogenicity. *Pseudomonas syringae* with more than 50 pathovars can infect several plants. On the other hand, *Pseudomonas putida* is a soil heterotrophic bacterium without pathogenic features. *Pseudomonas tolaasii* is a mushroom pathogen. *Pseudomonas fluorescens* is a broccoli pathogen also used in biocontrol processes since it protects certain plants against fungi and nematodes. Again, a plethora of substances are secreted and the composition of the outer cellular envelope accounts for this behavior. Cyclic lipopeptides (CLP) are among these substances expelled by *Bacillus* and *Pseudomonas cells*. Excellent reviews covering all issues related to those compounds have been published [8]–[10]. Over the last sixty years, four different families of nonribosomal cyclic lipopeptides were identified in the genus *Bacillus*. They are the surfactins, the iturins, the lichenysins and the fengycins [11].The large amount of data collected on CLP do not show any clear connection with animal or insect pathogenicity *in vivo*. In contrast, effects of CLP on cells, from bacteria to mammalian cells, are widely documented: they can act as detergents, antibiotics or cytotoxic, immunosuppressant or antitumor agents on a broad range of susceptible cells. The ultimate action mechanism accepted for these substances involves the interaction with the lipid bilayer hence converting a closed structure owning a fine tuned permeability, as is the case of a cell membrane, into a sieve which enables transmembrane uncontrolled fluxes. Notwithstanding, though this interaction may result from the hydrophobic hydrocarbon tail of the CLP molecular structure, few data are available on the role of the cyclic peptide moiety. Apparently, about ten aminoacids residues with variable sequence and nature form the lactone ring of CLP mixture produced by a *B. megaterium* strain [12]. The antibiotic activity of the CLP mixture completely vanishes after alkaline hydrolysis *in vitro* [12]. If the lactone ring of these substances is opened, inactive linear structures result [12]. The loss of antibiotic activity was also observed by treating surfactin with an enzyme of the tripeptidylaminopeptidase family produced by *Streptomyces* sp Mg1 [13]. Other lactonases related to the hydrolysis of n-acylhomoserine lactones, a class of quorum sensing inducers, are the paraoxonases (PON). These enzymes, produced by the PON gene family also in humans [14], may be putative inactivators of n-acyl homoserine lactones and cyclic lipopeptides from several bacterial sources thus acting as a defense mechanism against human infection by opportunistic bacteria.

Another point which seems to be of lesser interest when considering the action mechanism of CLP on susceptible cells, refers to the traffic of the whole molecule through the thick peptideglycan wall that surrounds Gram positive bacteria. Usually, researches on the action of antimicrobial peptides and CLP are focused on the interaction with the phospholipid cell membrane or model bilayers [15]. Most investigations specifically refer to the interaction between the antimicrobial peptides and the phospholipid cell membrane or model bilayers [16]. A lipopeptide produced by *Bacillus subtilis* has anti-tumor activity on LoVo cells [17] and a cyclic lipopeptide from *Bacillus natto* T-2 induces apoptosis in human leukemia K562 cells [18]. At the critical micelle concentration or above, it has recently been reported a major lytic effect on melanoma cells by the lipopeptide biosurfactant pseudofactin II [19]. Major problems encountered while trying to apply antimicrobial peptides *in vivo* have been their hydrolysis by proteases and their selective immune response [20]. Cyclic lipopeptides, similarly to lipopeptoids, are resistant to proteases due to their peculiar structural features such as their cyclic moiety and the high frequency of D-aminoacids residues in their backbone [13]. Here we propose that the bulky cyclopeptide head acts as a pathfinder. It presumably leads isolated CLP molecules towards the lipidic membrane bilayer by crossing the heavily polar and charged entourage of the peptideglycan. To do this, the cyclic nature of the peptide provided by the lactone ring must be intact so that the number of conformations assumed by the ring remain restricted to a few or a single conformation. Also the pH is found to play an important role because glutamic, aspartic and ornitine ionized residues could allow the transit athwart the peptideglycan restraint. The present results evidence the importance of the cyclic moiety for determining the antibiotic activity on susceptible *Bacillus cereus* cells, the resistance of CLP to proteases and the physical properties of the large secreted aggregates, shown to be micelles, which are formed from the balance between attractive interactions between hydrophobic tails and electrostatic repulsion between the cyclic peptide polar heads. The aggregates are inactive per se at the pH of the culture medium which is around 6–7. The knock out of the sensitive cells only takes place when the micelles are disassembled at pH>7 due to a high negative charge.

## Materials and Methods

### Bacterial strains, culture media and procedures for obtaining the cyclic lipopeptides mixture

Bacteria used in this study were isolated from a soil sample collected at the University of São Paulo campus. The morphological and biochemical characterization was previously described [12]. According to these data, the bacterium closely resembles *B. megaterium*. Identification service of the American Type and Culture Collection, Manassas, VA, USA recognized it as a *B. megaterium* strain (Project Report SC 3488), which throughout this work was named *B. megaterium* pL6. The composition of 500 ml of a minimal medium for efficient growth of *Bacillus* species included 0.8% MgSO_4_, 10 ml; 5 M NaCl, 17.0 ml; 1 M KCl, 3.4 ml; 85% H_3_PO_4_, 0.2 ml; 2% asparagine, 12.5 ml; 2% NH_4_NO_3_, 2.5 ml; 0.05% FeSO_4_, 3 ml; 20% glucose, 25.0 ml [12]. All stock solutions were sterilized individually and added in the specified volumes up to 350 ml of deionized water in the same order shown above. This is important to avoid precipitation. The pH was set to 7.0 with 1 M Tris –HCl, pH 8.0 sterile solution. Volume was complemented up to 500 ml with deionized water. Culture conditions and preparation of the mixture of biosurfactants produced by *B. megaterium* pL 6. Lyophilized cells of *Bacillus megaterium* pL6 were added to rich culture medium (1 mg cells to 20 ml of Luria Broth (LB) medium) and incubated for 12 h at 37°C, 200 rpm. This pre-culture was used to inoculate 500 ml of mineral medium in a 2 L Erlenmeyer flask where the new culture was incubated for 20 h at 37°C, 200 rotations per minute (rpm). Cells were centrifuged at 6,000 rpm (5,800×g) for 10 min in a Hitachi RPR9–2 rotor, the supernatant transferred to a 1 L flask and precipitated overnight at pH 2 with 5N HCl at 4°C. Centrifugation at 6,000 rpm (5,800×g) for 15 min in the RPR9–2 rotor recovered a precipitate which was extracted with 50 ml of pure ethanol for 3–4 h. The solid material resulting from the extraction was discarded after centrifugation at 10,000 rpm (12,000×g) in a Hitachi rotor RPR20–2. The supernatant from this centrifugation was evaporated at 50°C under vacuum, until an oily residue appeared at the bottom of the flask. The residue became soluble by adding 5 ml of deionized water at pH adjusted up to 8.0 with 2 M NaOH. It was transferred to a dialysis bag prepared with a membrane of 10 kDa cut off, previously boiled in a 2% sodium bicarbonate, 1 mM EDTA, pH 8.0 solution and exhaustively dialyzed against deionized water. The bag content was transferred to a 100- ml Erlenmeyer flask where it was lyophilized and stored under vacuum in a desiccator flask at −20°C.

### Determination of phosphorus content in the biosurfactant mixture

In order to ascertain the eventual presence of phosphorylated compounds (eg, phospholipids) in the biosurfactant mixture, the content of phosphorus was assayed in accordance with a previously described protocol [21]. Briefly, the previously weighed biosurfactant powder was diluted in pure water and aliquots of this solution were analysed for Pi content against a standard calibration curve of absorbance at 795 nm as a function of Pi nanomols (constructed with 0, 10, 30, 50, 70, 90 and 100 nanomoles of sodium monohydrogenphosphate) [21]. The results were expressed as Pi nanomols per mg of biosurfactant mixture.

### Determination of withdrawal of antibiotic components of the cyclic lipopeptide mixture by sensitive cells of *Bacillus cereus*

The rational of this assay is: the mixture of cyclic lipopeptides is added to *B. cereus* sensitive cells in a culture medium. Substances with antibiotic activity in the culture medium have to adhere to the cells for acting as antibiotics. Centrifugation of cells carries part of the active substances lowering its concentration in the medium. This should now be less active against these sensitive cells. In principle it should be noted that decrease in diameter of inhibition zone must be proportional to the adhesion of antibiotics to cells. The conditions described here allow observe this fact with reproducibility.

A 2 mL culture of *Bacillus cereus* was grown for 5 h up to early stationary phase at 37°C (∼4×10^7^ cells/mL). Then, 6.7 mg of the cyclic lipopeptide mixture in 50 µL of deionized water, pH 8 were added to the culture which was incubated for a further period of 120 min, 37°C. At 30 sec, 15 min, 30 min and 120 min, 70 µL were taken at each time. Samples were centrifuged at 10.000×g in a bench refrigerated centrifuge in order to remove cells. From each supernatant, 60 µL aliquots were loaded onto holes in an agar-LB Petri dish in order to observe inhibition haloes. A control lacking *Bacillus cereus* cells was incubated for 120 minutes.

### Determination of the effect of washing *Bacillus cereus* cells with NaCl solutions after incubation with the cyclic lipopeptides mixture

*Bacillus cereus* was inoculated in 20 mL of Luria Broth (LB). Culture was grown up to early stationary phase (∼4 h at 37°C). Cells were harvested by centrifugation at 6000×g for 10 min in a RPR20-2 rotor of a Hitachi Himac refrigerated centrifuge and resuspended in 2 mL of fresh LB in order to concentrate the cells by ten times. A 1.5 mL volume of an ethanolic extract of the cyclic lipopeptide mixture prepared as described previously [12] was evaporated under mild hot air stream and diluted with 100 µL deionized water at pH 8.0. Two *B. cereus* cell concentrates were separately incubated at 37°C with 100 µL of the cyclic lipopeptide mixture prepared as above, for 30 sec and 45 min respectively in 2 mL eppendorf tubes. Two 70 µL samples were removed from each tube, centrifugated at 10.000× in a bench refrigerated centrifuge. The cell pellet was washed for 5 min in a vortex device with 200 µL of deionized water and resuspended in 70 µL of a 0.05 M NaCl or 0.4 M NaCl. Cells in 4 tubes were vortexed with saline solutions and centrifuged at 10.000×g. From each tube 60 µL samples were tested in agar-LB Petri dish to detect antibiotic activity against *B. cereus*. Two controls were done: a) A 1.5 mL volume of an ethanolic extract of the cyclic lipopeptide mixture prepared as described previously [12] was evaporated under mild hot air stream and diluted with 100 µL deionized water at pH 8.0 (this was mixed with 2 ml of LB and incubated for 45 min at 37°C; an aliquot of 60 µL was used in the same Petri dish to assay the whole antibiotic activity of the cyclic lipopeptide mixture); b) 60 µL of a 0.4 M NaCl solution were tested for antibiotic activity in the same agar-LB Petri dish.

### Determination of the pH effect on the antibiotic activity of the cyclic lipopeptide mixture

Four 1.5 mL samples of an ethanolic extract of the cyclic lipopeptide mixture were evaporated and taken up in water as described above. The pH in each sample was adjusted to 2, 4, 6 and 8 respectively in order to assess antibiotic activity in an agar-LB Petri dish. As control, 200 µL of deionized water were plated onto the same agar-LB Petri dish.

### MALDI-TOF mass spectrometry analysis of samples containing cyclic lipopeptide mixtures

Three kind of samples were analyzed by MALDI-TOF MS: whole cells, supernatants of cultures grown in mineral medium and preparations of aggregates made up by mixing ethanolic extracts with Milli Q water. Usually, samples were mixed in several dilutions with a 10 mg/mL saturated matrix solution of *alpha*-cyano-4-hydroxycinnamic acid in acetonitrile: water 1∶1 containing 0.1% of trichloroacetic acid. Dilutions were 1∶1, 1∶2 and 1∶4. Whole cells were raised directly from colonies grown in agar-LB by touching with a sterile toothpick and washing in 1, 2 or 4 µL of the matrix solution. The dilution displaying the highest ionization signal was used for complete analysis of the mixture. Further details are: 0.5 µL were loaded onto the instrument metal plate, dried and examined in the positive mode in a Bruker Daltonics UltrafleXtreme MALDI TOF TOF mass spectrometer working with a nitrogen laser and 20 Kvolt ion accelerating potential.

### Determination of the lytic activity of a mixture of cyclic lipopeptides on cells of *B. cereus* before and after alkaline treatment

A 10 mg/mL solution in ethanol of the cyclic lipopeptide mixture was prepared from lyophilized material. Volume equivalent to 1.6 mg (160 µL) was evaporated and suspended in 160 µL of Milli Q water to which 40 µL of a 0.5 M KOH solution were added. The sample was heated up to 70°C for 4 hours after which it was neutralized with 5.5 N HCl. Two controls were prepared exactly in the same conditions except that in one control water was added instead 0.5 M KOH and pH was adjusted to 8 before use; the other control was prepared without the cyclic lipopeptide mixture but also neutralized with 5.5 N HCl. All the samples (4 µL) were loaded onto a confluent overnight *Bacillus cereus* culture grown at 37°C in agar-LB. Results were assessed after leaving Petri dishes for 12 h in a refrigerated chamber at 4°C.

### Particle sizing and zeta-potential analysis for the lipopeptides mixtures at pH 6–7

Size distributions, zeta-average diameters (Dz), and zeta potentials (ζ potentials) were obtained by dynamic light scattering (DLS) using a Zeta plus−Zeta potential Analyzer (Brookhaven Instruments Corporation, Holtsville, NY) equipped with a 677 nm laser with measurements at 90°. The polydispersity of the dispersions was determined by dynamic light scattering (DLS) following well defined mathematic equations [22]. Mean hydrodynamic diameters (mean Dz) were obtained from the log-normal distribution of the light-scattering intensity curve against the diameter. ζ potentials were determined from the electrophoretic mobility μ and Smoluchowski equation ζ = µη/ε, where η and ε are the viscosity and the dielectric constant of the medium, respectively. The measurements were performed after thermal equilibrium at 25±1°C. Zeta potential values were measured in 1.5 mL of *Bacillus megaterium* pL 6 mineral medium culture supernatants before or after dialysis. Since *B. megaterium* consumed most of the salt components of the culture medium after 24 h growth, the final ionic strength falled to values lower than 10 mM monovalent salt so that the zeta-potential could be determined without damage to the apparatus electrode. Data were obtained from 10 runs of 5 cycles. For size determinations, 3 mL samples were prepared and analyzed by 10 runs of 5 min. each. Only data displaying baseline higher than 7 were selected for further analysis.

### Determination of the osmotic response for the biosurfactant aggregates at pH 6–7

In order to ascertain the existence of an inner aqueous compartment in the biosurfactant aggregates at pH 6–7, osmotic gradients were established by adding a hypertonic D-glucose solution to the biosurfactant aggregates and following the kinetics for Dz (nm) as a function of time. In response to and hypertonic medium, for aggregates of the vesicular or liposomal type, a decrease in size with time should be expected due to vesicle shrinkage. For massive aggregates of the micellar type, the size should remain constant.

## Results and Discussion

**Figure 1:**
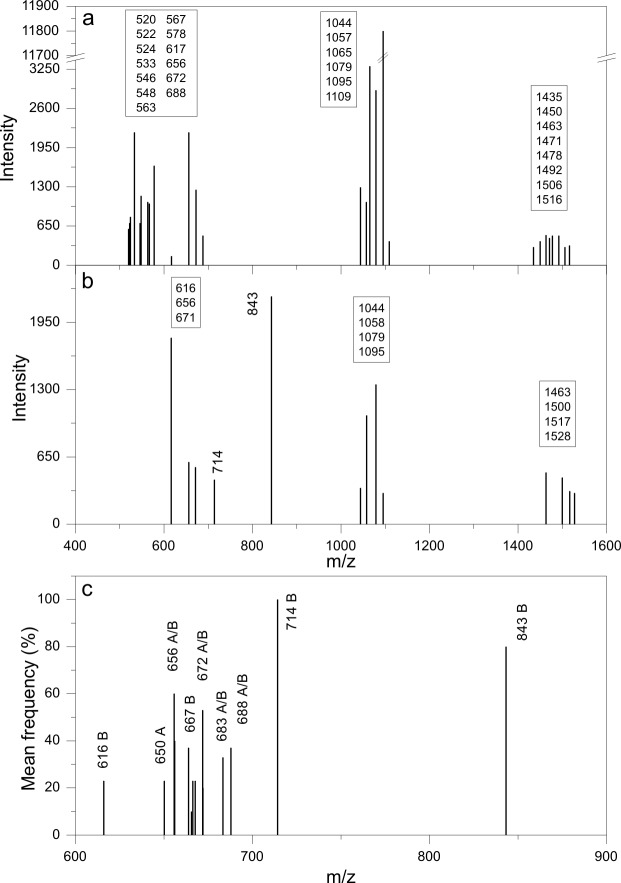
MALDI-TOF mass spectra of cyclic lipopeptides from *Bacillus megaterium* pL6 and other *Bacillus* species. The spectra for CLP secreted in the culture medium by *Bacillus megaterium* pL6 as a mixture of surfactins, iturins, lichenysins and fengycins and other low molecular weight substances (600–900 m/z) (a) are also found in intact cells (b). In (c), more than 30 spectra from culture supernatant (labeled A) and whole cells (labeled B) from several *Bacillus* (*B. megaterium*, *B. cereus*, *B. lentus*, *B. laterosporus*, *B. polymyxa*, *B. circulans*, *B. pumilus*) were analyzed to obtain the frequency of MALDI-TOF MS peaks versus average m/z of low molecular weight substances (600–900 m/z). The ion 714 m/z was detected practically in all the spectra from whole cells but not found in the supernatant as expected for a cell wall component; compounds found both in the cell culture supernatant and detected in whole cells are assumed to be exported substances (c).

The MALDI-TOF mass spectrum of a CLP mixture isolated from the supernatant of a late stationary phase culture of *B. megaterium* (Figure 1a) is similar to the one obtained for intact cells (Figure 1b). These two spectra however, are different in the sense that the exposition of substances in solution to the laser beam allows their proper ionization but, in the cell, they may not become ionized. The peaks in Figure 1b probably are due to exposed substances at the cell wall surface which are readily ionized by the laser beam. MALDI-TOF MS spectra from intact cells are possibly composed of ions derived from some cell envelope components [23]. Probably, the efficiently ionized substances having high signal intensity are the most exposed to the laser beam. Those released to the culture medium by the bacillus are likely quite exposed whereas those in the cells are the ones caught by the laser beam on the verge of being secreted to the medium. The substances having molecular weights in the 900–1600 m/z range were identified as exported CLP that are always detected both in intact cells and the culture supernatant. Figure 1c shows the gathering of 30 mass spectra from *Bacillus megaterium* pL6 and other *Bacillus* species over the 500–850 m/z range with peaks systematically detected in the culture supernatant (labeled as A) and in the washed cells (labeled as B). One should notice that some peaks were detected both for the supernatant samples and the washed cells (A/B). The 655, 656, 664, 671, 688 m/z peaks were obtained both for the supernatant and the cells whereas the 714 and 843 m/z peaks were detected for intact cells only. All these compounds were thought to be lipids due to their behavior in the extraction procedure. For tentative identification, the Lipid Molecular Structure Database (http://www.lmsd.tcd.ie/) was queried suggesting their glycerophospholipidic nature with different head groups. The CLP mixture quoted throughout this work possibly contains a set of these low molecular weight substances. Furthermore, the quantitative determination of phosphorus content for CLP lyophilized samples yielded 176 nanomols of Pi per mg indicating that this may be a correct assumption.

Panel c on Figure 1 is important to show the peaks that are available from different bacteria species and that appear both in the cells (probably in the cell envelope) and in the secreted compounds. The MALDI-TOF technique applied to the cells can only ionize substances that are peripherically located on the cell. Therefore, the detected peaks in the cells must correspond to substances that are in the cell envelope. Conversely, peaks that appear in the cells (eg, the 714 and the 843 m/z peaks in Figure 1b) and do not appear in the supernatants (Figure 1a) are supposed to belong to the cell wall structure.

**Figure 2:**
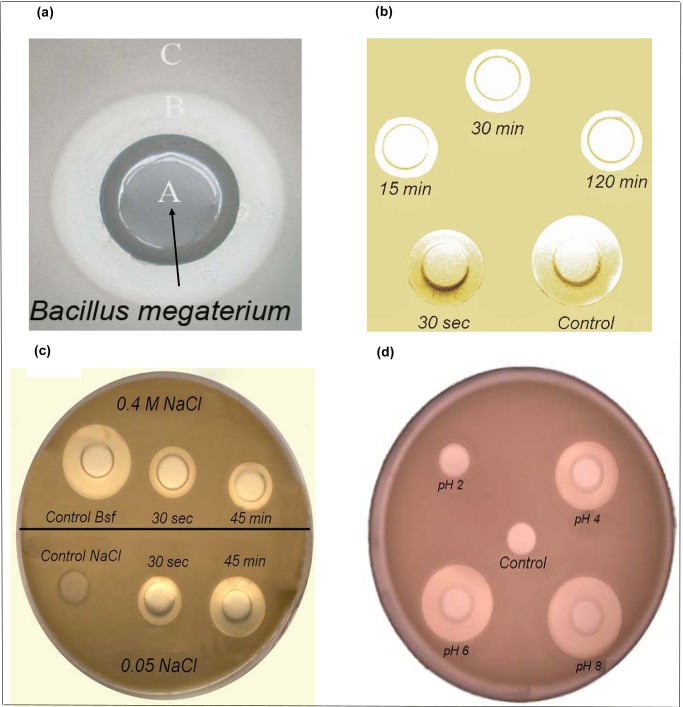
Inhibition halos and lytic activity of CLP from *Bacillus megaterium pL6* against *Bacillus cereus.* On panel (a), the inhibition halo B against *Bacillus cereus* confluent layer C is induced by *Bacillus megaterium* pL6 cultivated inside the hole A. On panel (b), the withdrawal of the antibiotic activity produced by *B. megaterium* by cells of *B. cereus* is a function of the interaction time (50 µL of a cyclolipopeptides solution at 10 mg/mL were incubated at 37°C with 2 mL stationary cell culture of *B. cereus* for 15, 30 and 120 minutes before withdrawing 70 µL samples for centrifugation and determination of supernatant residual activity against *B. cereus* from inhibition haloes); the control was performed in the absence of *B. cereus* cells. On panel (c), the withdrawal of the antibiotic activity from *B. cereus* is seen in the presence of two different ionic strengths (0.05 and 0.4 M NaCl). *Bacillus cereus* cells were grown up to stationary phase, concentrated 10 times before adding 100 µL of 10 mg/mL cyclolipopeptides solution at pH 8.0 to 2 mL of the concentrated *B. cereus* suspension LB for 0.5 or 45 minutes interaction; thereafter mixtures were centrifuged and resuspended in water for removal of biosurfactant excess; 70 µL of the pelleted cells were resuspended in the NaCl solutions and centrifuged again before withdrawal of 60 µL of each supernatant for determining the inhibition haloes against *B. cereus* after incubation for 9 h at 37°C. Controls were 60 µL of the Bsf solution (10 mg/mL) or 0.4 M NaCl solution only. On panel (d), the pH of the biosurfactant solution affects the inhibition halo of *B. cereus*. The pH was adjusted to 2, 4, 6 and 8 by adding 1N HCl or 1N NaOH to 0.2 mL of biosurfactant solutions before loading 0.1 mL of each solution onto the holes of a Petri dish containing *B. cereus*; the control was 0.1 mL milli Q water adjusted to pH 2.

Figure 2 (a) exhibits the inhibition halo induced by *Bacillus megaterium* pL 6 cells against a highly sensitive *Bacillus cereus* strain. The Petri dish was mounted with two layers of Luria Broth agar (LB) with 1.5% agar at the bottom and 0.8% agar on the top. The hole in the plate center (A) in the top layer was seeded with 0.1 mL of a fresh suspension of *B. megaterium* pL 6 cells cultured up to the stationary phase. The top agar layer surrounding the central hole was previously mixed with 0.3 mL of a fresh stationary *B. cereus* LB culture. After incubation at 37°C overnight, a visible inhibition halo (B) contrasted with the growth of *B. cereus* in the region (C) of the Petri dish. The inhibition halo indicates that *B. megaterium* pL6 cells release substances which diffuse through the agar preventing the growth of *B. cereus*. The radial diffusion occurs roughly in two dimensions, differently from the tridimensional process taking place in nature when species of microorganisms compete for the same ecological niche [24]. Figure 2 (b) shows the withdrawal of the CLP by *B. cereus* over a range of interaction times. Increasing the interaction time between CLP and *B. cereus* cells, leads to a reduction of CLP in the supernatant as visualized by the reduction of the inhibition halo diameter. From 15 minutes interaction time, the limiting withdrawal of the CLP by the cells is achieved as seen from the constant halo diameter from 15 up to 120 minutes. In Figure 2 (c), the release of CLP with time at two different ionic strengths (0.05 and 0.4 M NaCl) increases with time at 0.05 M NaCl and remains constant with time at 0.4 M NaCl. At 30 s of interaction time between *B. cereus* and CLP, CLP release from the cells occurs for the two different ionic strengths. However, at 45 minutes of interaction time and 0.05 M NaCl, CLP release is larger than the one induced by the 0.4 M NaCl solution. The release of antibiotic activity is less efficient at high ionic strength, suggesting the occurrence of CLP/cell interactions different from the electrostatic one which should be screened in accordance with the magnitude of the ionic strength. In agreement with observations made for certain lipoplexes, which are lipid-DNA complexes intended to transfect DNA into eukaryotic cells, a high salt concentration triggers some lipid-DNA dissociation although the vast majority of the complexes remain as such [25], possibly due to the hydrophobic interaction between lipid and DNA [26]. Probably, the electrostatic interaction is not the sole driving force of CLP into the cell envelope structures. Figure 2 (d) shows the increase in CLP activity against *B. cereus* with pH: CLP mixture is more active over the 7–8 pH range. The antibiotic activity detected against *B. cereus* cells may be related primarily to deprotonated carboxyls or negatively charged CLP. Ionizable groups in these CLP are: a) carboxyl from glutamic acid residues or aspartic acid residues; b) amino group from ornitine residues. Surprisingly, negatively charged bacterial cells interact with similarly charged CLP. However, local charges on the CPL molecules, varying from polar to fully charged groups depending on pH and on the group nature and polarizability [27], certainly play a crucial role on the interaction between the CLP and cells. These CLP molecules are doubtless, well equipped to disassemble the cell envelope of several kinds of microorganisms. Since both the cell wall and the CLP have a net negative charge, electrostatic interaction between CLP and cell wall may occur at local positively charged groups as is the case of protruding section of proteins embedded into the peptideglycan layer charged groups. The disassembly of CLPs themselves due to dissociation of negatively charged moieties at pH 6–8 and intermolecular electrostatic repulsion presented in this work later on further supports this explanation for the increase in activity of the non aggregated CLP molecules.

**Figure 3.The:**
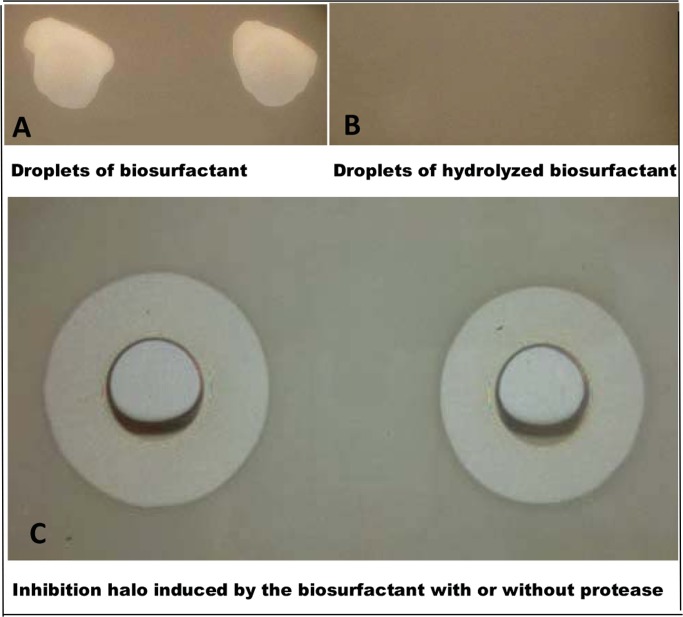
The cyclic moiety of the lipopeptides is essential for their lytic activity against *B. cereus* and resistance to proteases. On the upper left panel, two samples (80 µL each) of the biosurfactant mixture (16 mg/mL) in Milli Q water were loaded onto a confluent pre-growth *Bacillus cereus* agar culture. On the upper right panel, the biosurfactant mixture was pretreated for alkaline hydrolysis with 5N KOH for 4 h at 70°C, neutralized with 5 N HCl and tested against *B. cereus*. On the lower panel, the resistance of the biosurfactant mixture to a protease from *B. amyloliquefaciens* at a final concentration of 5 mg/mL (37°C/5 h) can be appreciated.

On Figure 3, panels A and B indicate that CLP causes lysis on *B. cereus* cells and that this activity depends on the integrity of the CLP cyclic moiety, respectively. The strong alkaline treatment to which the CLP mixture was submitted disrupted the lactone ring [12] and abolished its biological activity (Figure 3B). Figure 3C depicts the resistance of the CLP mixture to proteases so that its biological activity understood as the one conferred by the cyclic moiety is maintained due to the resistance of the peptide ring to be recognized and opened by proteases. This is possibly achieved by conformations which severely twist the peptide cycle thus hiding peptide bonds and/or by the occurrence of D -aminoacids residues in the peptide backbone.

**Figure 4:**
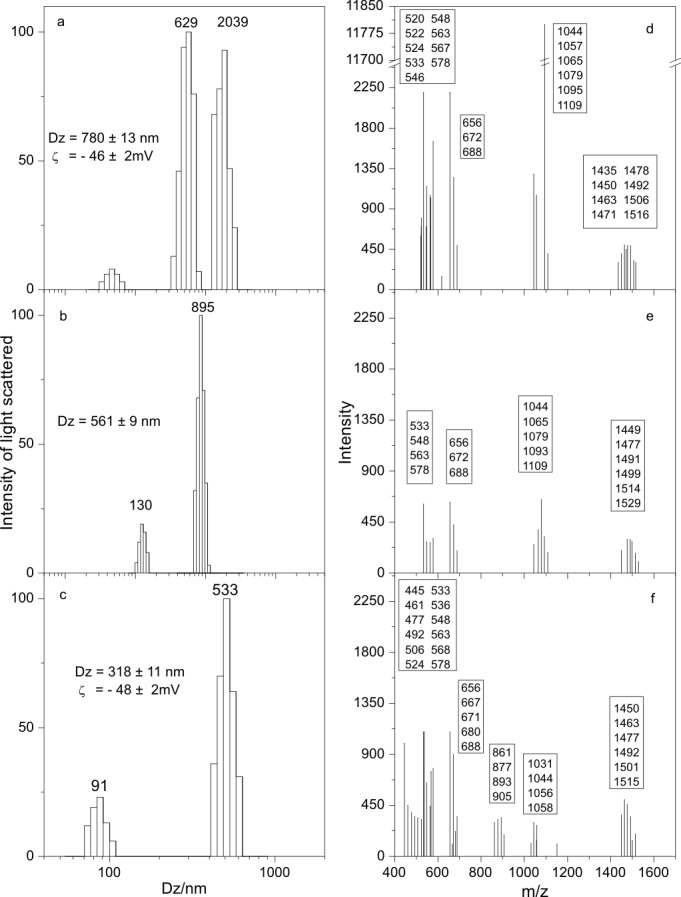
Cyclic lipopeptides assemble as negatively charged aggregates with size distributions and mean sizes that change with sample processing exhibiting nevertheless very similar composition from MALDI-TOF mass spectra. Size distribution, mean hydrodynamic diameter (Dz) and zeta-potential from dynamic light scattering (a, b, c) and MALDI-TOF mass spectra for the *B. megaterium* culture supernatant (d, e, f) for the cell free, late stationary phase culture supernatant before (a, d) and after exhaustive dialysis against Milli-Q water (b, e) or after dialysis plus centrifugation at 17.000×g for 10 minutes (c, f).

In Figure 4, dynamic laser light scattering by fresh *B. megaterium* supernatants reveals particles having mean diameter up to ∼780 nm, a size consistent with large aggregates which were formed spontaneously in a mineral culture medium made up from salts, nitrogen and carbon sources (originally at pH∼8 but changing to lower values, ∼6–7, after growth to the late stationary phase). The aggregate size decreases after dialysis of the supernatant (compare panels a and b on Figure 4) as well as after dialysis and centrifugation at 17.000×g from ∼780 to 318 nm (compare panels a and c on Figure 4). The zeta potential, which measures the electrical potential at the shear plane in the particle neighborhood, is −46 mV (Figure 4a) and −48 mV (Figure 4c), barely changing after dialysis and centrifugation and showing a good colloidal stability for the negatively charged CLP aggregates. Figure 4 exhibits also MALDI-TOF MS of the dispersions both before (Figure 4d) and after dialysis against water or minimal medium (Figure 4e). They are qualitatively similar displaying the whole set of substances. After dialysis, all the peaks are retained by the dialysis membrane indicating aggregate formation. If after dialysis the suspension is centrifuged at 17.000×g, the supernatant still displays a similar composition (Figure 4f) suggesting aggregates small enough to remain in the supernatant after being centrifuged or alternatively, a set of disassembled substances at a concentration which is lower than the critical micelle concentration (CMC) and thus are not forming aggregates. The spontaneous assembly of CLP and low molecular weight lipids raises the question of what could be the role of such structures in the antibiotic activity. Likely, the aggregation concentrates active molecules in enormous particles in order to maximize the attack against the cell envelope. This inference may also be applied to other *Bacillus* species since they export a similar set of substances to the culture media (results not shown). Since the CLP mixture is secreted into a mineral culture medium having several inorganic salts and hence a relatively high ionic strength at the pH of water (pH 6.3), it is a reasonable assumption that from the CMC threshold, aggregates are formed. These, by effect of the ionic strength, could increase in size due not only to the screening of charges on the micelle ionic heads, but also due to the salting out effect on the hydrocarbon chains [28].

**Figure 5:**
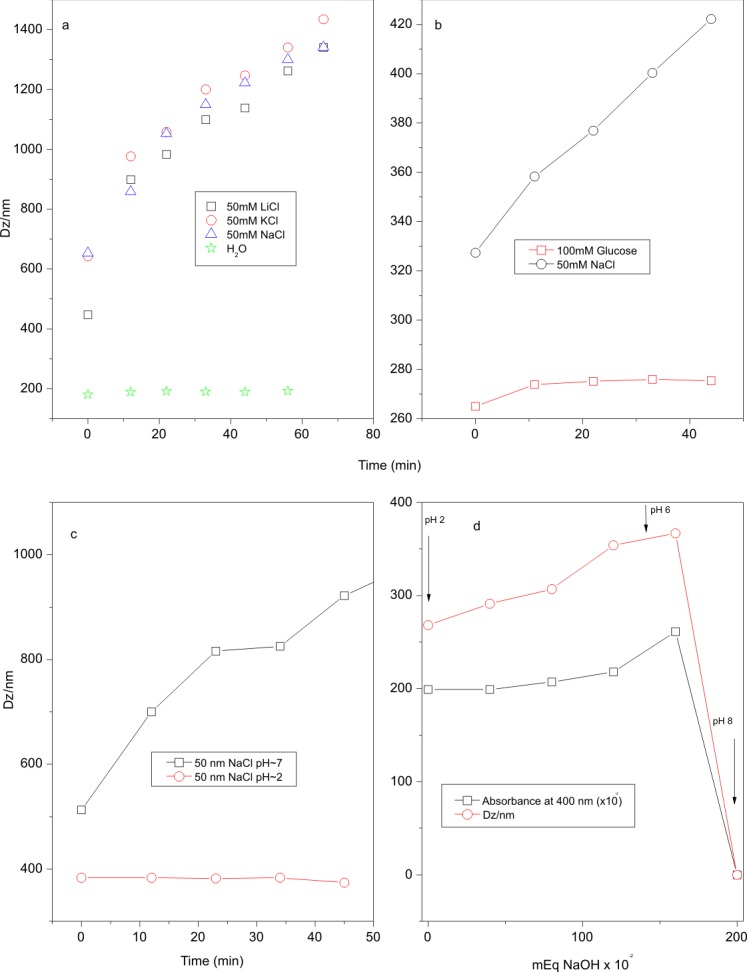
The CLP aggregates change their size with the environmental conditions such as ionic strength and pH but are not responsive to osmotic gradients evidencing their massive nature and the absence of an internal aqueous compartment. Effects of salt (a), osmotic gradient (b) and pH (c) on the kinetics of zeta-average diameter (Dz) of biosurfactant dispersions after adding different media. The effect of changing pH from 2 to 6 or to 8 on Dz and absorbance at 400 nm of the biosurfactant dispersion is shown in (d). Ethanol extracts of the CLP mixture (0.2 mL) were added of 3 mL water solutions (a, c) or 0.2 mL of ethanolic CLP were added of 4 mL of water solutions (b, d) forming a suspension of milky, opalescent appearance. The mixture was gently mixed until the water addition was complete.

CLP ethanol extracts form turbid suspensions when mixed with Milli -Q water. Typically, adding the CLP ethanol extract to water solutions of monovalent salts (50 mM sodium salts) leads to an increase in particle size which could be determined by dynamic laser light scattering (Figure 5, panel a). Irrespective of the counterion ionic radius (Li+, K+, Na+), the ∼500 nm aggregates increased their diameter up to 1500 nm after 60 min. On panel b (Figure 5), the absence of osmotic response by CLP aggregates upon addition of a 100 mM D-glucose solution is shown. For closed vesicular structures, acting as semipermeable barriers, one should expect shrinkage and reduction in size induced by the hypertonic medium [29], [30]. On panel c (Figure 5), the CLP aggregation induced by a 50 mM NaCl solution is shown to depend on the pH being absent at acidic pH values such as 2.0. At pH∼7, when carboxyl and phosphate groups are deprotonated and negatively charged, the screening effect of 50 mM NaCl induces further CLP aggregation and increase in particle size (panel c on Figure 5). At pH∼2, the quoted groups are protonated and further aggregation upon addition of NaCl does not occur (panel c on Figure 5). To further assess the influence of pH on the aggregate size, a titration of CLP aggregates (starting at pH 2) with 50 mM NaOH was performed up to pH 8 as shown on the panel d of Figure 5. Both the turbidity at 400 nm and the mean particle size (Dz) remained relatively constant over a range of low pH values but over the 6–8 pH range, both turbidity and size of CLP aggregates abruptly decreased indicating the disaggregation of the CLP assemblies. Possibly, at the moderate ionic strength employed, the dissociation of negatively charged carboxylates and phosphates in the CLP molecules takes place over the 6–8 pH range inducing an increase in the intermolecular electrostatic repulsion and disassembly of the aggregates. Consistently, the antibiotic activity of the lyophilized CLP mixture solubilized in water against *Bacillus cereus* cells only occurs around pH 6–8 [12] when the CLP dispersions change from a turbid to a transparent and clear appearance in aqueous solution (panel d on Figure 5).

**Figure 6:**
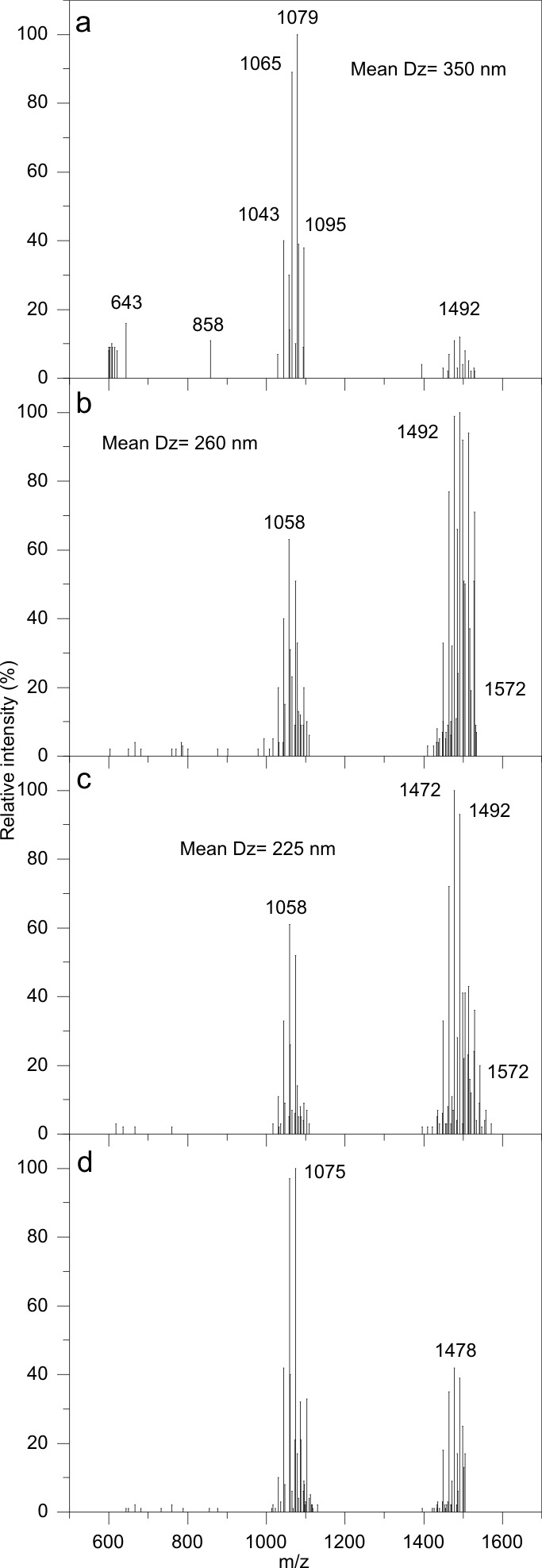
Dialysis and centrifugation barely affect the composition of the cyclic lipopeptides mixture depicted from MALDI-TOF mass spectrometry. MALDI-TOF MS of CLP aggregates with mean hydrodynamic diameters (Dz) determined by DLS for CLP in ethanol (0.2 mL) added of a 50 mM NaCl water solution at pH 6.3 (4 mL) before (a) and after dialysis (b). On panel (c), the mass spectrum for the supernatant of the same sample shown on panel (b) after centrifuging (17000×g/10 min.). On panel (d), the MS for the precipitate obtained after centrifuging (17000×g/10 min.) the sample used in (b).The relative intensities are shown for each molecular ion (m/z) detected.

Figure 6 shows MALDI-TOF MS for the various substances of the CLP ethanol extract added of a 50 mM NaCl solution before (panel a) and after dialysis (panel b). After dialysis, further centrifuging the CLP dispersion to separate the supernatant (panel c) and the precipitate (panel d) yielded similar mass spectra. The substances corresponding to the peaks at 1081, 1095 and those within the 640–900 m/z range remain all inside the dialysis bag after the dialysis procedure as seen from the comparison between panels a and b on Figure 6. After dialysis and centrifugation, the profile of the remaining peaks is not substantially changed in the supernatant (panel c on Figure 6) or even in the precipitate (panel d on Figure 6), which conclusively indicates that several CLP are forming the aggregates remaining in the dialysis bag after dialysis. Mean hydrodynamic diameters obtained after each procedure are quoted in the subfigures and evidence a small decrease in size after dialysis or after dialysis and centrifugation. In general, Figure 6 indicates that dialysis and centrifugation barely affect the composition of CLP aggregates first formed and secreted by *B. megaterium* in the culture medium.

The results commented above suggest a mechanism by which the CLP mixture may act on bacterial sensitive cells destroying them both in the culture medium and other natural environments.

CLPs act by a mechanism substantially less sophisticated than most of the so called antibiotics. They interact with the membrane lipid bilayer producing pores [31] and disrupting it extensively, as postulated in the carpet model [32]. Another class of substances having similar behavior are the antimicrobial peptides (AMPs), evolutionarily conserved components of the innate immune response and found among all classes of life. The mammalian innate immune system acts before the specific immune response is triggered and is mediated by small hydrophobic peptides able to disrupt the cell bilayer membrane. Fundamental differences exist between prokaryotic and eukaryotic cell envelopes representing differential targets for AMPs. These cationic peptides normally bind more strongly to anionic bacterial membranes than they do to mammalian membranes [33], [34]. Before reaching the lipid bilayer, CLPs must traverse a complex molecular barrier consisting of small or bulky hydrophobic, polar or charged groups which are attached to the bilayer, or to proteins or belong to the peptide glycan layer in bacteria. It is difficult to envisage how CLPs having mostly hydrophobic properties can achieve its target through such an environment. CLPs own a hydrocarbon hydrophobic tail linked to a polar peptide moiety which may assume a rather restricted range of conformations due to the lactone or lactam rings. Conformational constraints are often linked to the biological activity. A conformational lock provided by the peptide cyclization, amino acid nature and sequence, chirality, intra-molecular hydrogen bonding, β turns and overall cycle conformation may be directly related to biological activity even when receptors are not involved as is the case of the CLPs.

CLPs forming mixtures with low molecular weight compounds such as phospholipids, surfactins, iturins and fengycins assemble in large aggregates of the micelle type acting as concentrators of active molecules in a pH∼6 environment. These aggregates traverse the highly polar cell wall envelope of Gram (+) bacteria until chargeable groups of the cyclic peptide head change their pKa in order to mimic conditions observed at pH∼8, in which micelles disassemble in components now free to interact with the lipidic bilayer via driving forces such as the hydrophobic effect, the dipole-dipole interaction, hydrogen bonds and others.
